# Increased Pro-Inflammatory Cytokines, C-Reactive Protein and Nerve Growth Factor Expressions in Serum of Patients with Interstitial Cystitis/Bladder Pain Syndrome

**DOI:** 10.1371/journal.pone.0076779

**Published:** 2013-10-17

**Authors:** Yuan-Hong Jiang, Chung-Hsin Peng, Hsin-Tzu Liu, Hann-Chorng Kuo

**Affiliations:** 1 Department of Urology, Buddhist Tzu Chi General Hospital, Hualien, Taiwan; 2 School of Medicine, Tzu Chi University, Hualien, Taiwan; 3 Department of Urology, Taipei Tzu Chi Hospital, Buddhist Tzu Chi Medical Foundation, New Taipei City, Taiwan; 4 Department of Toxicology, School of Medicine, Tzu Chi University, Hualien, Taiwan; University of San Francisco, United States of America

## Abstract

**Objective:**

The etiology and pathogenesis of interstitial cystitis/bladder pain syndrome (IC/BPS) are unclear. Chronic inflammation is considered the main pathology of IC/BPS. This study measured the serum c-reactive protein (CRP), nerve growth factor (NGF) and pro-inflammatory cytokine/chemokine interleukin (IL)-1β, IL-6, tumor necrosis factor (TNF)-α, and IL-8 expression in patients with IC/BPS to elucidate the involvement of systemic inflammation in IC/BPS.

**Methods:**

Serum samples were collected from 30 IC/BPS patients and 26 control subjects. The concentrations of serum nerve growth factor (NGF), IL-1β, IL-6, TNF-α, and IL-8 were quantified using a bead-based, human serum adipokine panel kit. Serum C-reactive protein (CRP) was also assessed. Differences of serum CRP, NGF, IL-1β, IL-6, TNF-α, and IL-8 levels between the IC/BPS patients and controls were compared, and correlations between CRP and pro-inflammatory cytokines and chemokine were also evaluated.

**Results:**

The results showed that CRP level (*p* = 0.031), NGF (*p* = 0.015) and pro-inflammatory cytokines/chemokine IL-1β, IL-6, TNF-α, and IL-8 levels were significantly higher in the patients with IC/BPS than among controls (all *p*<0.001). Significant associations were observed between IL-1β and IL-8 (*p*<0.001), IL-6 and CRP (*p* = 0.01), IL-6 and IL-8 (*p* = 0.02), and IL-6 and TNF-α (*p* = 0.03).

**Conclusion:**

Increased pro-inflammatory cytokines/chemokine (IL-1β, IL-6, TNF-α, and IL-8) expression in the sera of IC/BPS patients implies not only mast cell activation, but also that other inflammatory mediators play important roles in the pathogenesis of IC/BPS. Thus, for some patients, IC/BPS is considered a chronic inflammatory disease.

## Introduction

Interstitial cystitis/bladder pain syndrome (IC/BPS) is a syndrome characterized by urinary urgency and frequency, usually with pelvic pain and nocturia, in the absence of bacterial infection or identifiable pathology [1]. Although there are many theories, the etiology of IC/BPS remains obscure. There is a lack of consensus on the pathophysiology of IC/BPS. The chronic pain symptomatology in IC/BPS may be due to persistent abnormalities in the urinary bladder, which activate the afferent sensory system and central nervous system sensitization [2]. Recent findings suggest several pathophysiological mechanisms including epithelial dysfunction, activation of mast cells, neurogenic inflammation, autoimmunity, and occult infection [3]. The diagnosis of IC/BPS solely made by clinical and cystoscopic hydrodistention and exclusion of other bladder disorders might no longer be adequate [4]. Development of urine or serum biomarkers that can be used in conjunction with clinical symptoms might increase the rate of early and accurate diagnosis of subtypes of IC/BPS.

Several previous studies have linked IC/BPS to chronic inflammation of the urinary bladder. Nerve growth factor (NGF) in urine and in bladder tissue and serum C-reactive protein (CRP) all increase with IC/BPS [5]–[7]. Previous studies also showed that the immune system might play an important role in the pathophysiology of IC/BPS [8].

The clinical symptoms of IC/BPS and overactive bladder syndrome (OAB) are similar. Recent investigation of urinary chemokines in OAB patients showed increased monocyte chemotactic protein-1 and inflammatory proteins compared to controls patients [9]. In addition, IC/BPS involves an aberrant differentiation program in the bladder urothelium that leads to altered synthesis of several proteoglycans, cell adhesion, tight junction proteins, and bacterial defense molecules such as GP51 [10]. These findings led to the rationale for searching for potential clusters of urinary biomarkers to detect IC/BPS in patients with frequency urgency syndrome.

IC/BPS is a heterogenous syndrome with different severity of inflammation. Urinary inflammatory mediators have been investigated as noninvasive markers to identify IC/BPS. Urinary IL-6 levels was previously shown to elevate in IC patients with severe inflammation and are positively associated with pain scores [11]. Both IL-6 and IL-8 urine levels are elevated in patients with active IC, but similar levels are also found in patients who met or did not meet the National Institute of Diabetes, Digestive, and Kidney Diseases criteria [12]. A study comparing urinary IL-6 between IC/BPS and control patients found that sensitivity was 70%, and specificity 72.4% for the diagnosis of IC [13]. However, a recent study suggested that IL-8 is an important normal urothelial growth factor and is necessary for normal urothelial cell survival in vivo and in vitro. Lower IL-8 expression levels may contribute to pathophysiology of IC/BPS [14]. This controversial evidence indicates that a single urinary cytokine level might not be useful in the diagnosis of IC/BPS.

In this study, we measured the levels of serum cytokines/chemokines in patients with IC/BPS in addition to CRP and NGF. Although most of these cytokines/chemokine have been found to be increase in the urine of patients and have proposed to be putative biomarkers useful in the diagnosis of IC/BPS, elevated serum cytokines/chemikines level in IC/BPS patients has rarely been reported. The results may provide evidence that systemic chronic inflammation is involved in IC/BPS patients.

## Materials and Methods

This was a pilot study of serum cytokines and chemokine in IC/BPS patients. Serum samples were collected from 30 consecutive IC/BPS patients and 26 control subjects. All patients were diagnosed with IC/BPS by characteristic cystoscopic findings after hydrodistention. Only non-ulcer type IC/BPS patients and patients without additional systemic/inflammatory disease (such as diabetes mellitus, arthritis, systemic lupus erythematosus, Sjogrene syndrome), neurogenic lesion or active urinary tract infection (UTI) were included in this study. The patients’ bladder pain score was measured by the visual analog of pain (VAS) scale. The control subjects were recruited from among hospital employees and research assistants who had no history of UTI and had no lower urinary tract symptoms. This study was approved by the Ethics Committee of the hospital (identifier: Tzu Chi General Hospital IRB 101–16). Written informed consent was obtained from every patients and control subjects before the blood was withdrawn.

Five to ten milliliters of blood was withdrawn and collected from each subject. Blood samples were allowed to clot on ice for 30 to 60 min and were then centrifuged in a swinging bucket rotor at 4°C, 3000 rpm for 15 min. The supernatant serum was carefully collected and stored at −80°C until the tests were performed. The concentrations of serum NGF, IL-1β, IL-6, tumor necrosis factor-α (TNF- α), and IL-8 were quantified using a bead-based human serum adipokine panel kit (Millipore, Billerica, MA, USA) according to the manufacturer’s instructions. For the NGF assays, duplicate samples and serial dilutions of the NGF standards were added to 96-well microtiter plates and incubated overnight. Data were analyzed using the LX 200™ platform (Millipore, Billerica, MA, USA). R^2^ of the standard curve equaled 1. Serum CRP was measured using the Cobas Integra 400 autoanalyzer running a particle-enhanced turbidimetric assay (Cobas Integra C-Reactive Protein Latex; Roche Diagnostics). Assays were performed at the Hualien Tzu Chi General Hospital Central Laboratory. The lower limit of detection was 0.10 mg/L.

Differences in serum CRP, NGF, IL-1β, IL-6, TNF-α, and IL-8 levels between the IC/BPS patients and the controls were compared using the Mann-Whitney U test for non-parametric data. Furthermore, Pearson’s correlation coefficients were calculated to ascertain correlations between serum CRP, pro-inflammatory cytokines and chemokines. In order to clarify the effect of age and gender on serum cytokines/chemokine levels, correlation of these pro-inflammatory proteins with age and gender was performed in both IC/BPS and control groups. The CRP, NGF and cytokines/chemokine levels were also correlated with the VAS scores in IC/BPS patients. The data shown in the results were expressed as mean plus and minus standard error of the mean (SEM). All analyses were conducted using SPSS for Windows (version 12, SPSS, Chicago, IL). Two sided *p*-value of less than 0.05 were considered significant.

## Results

A total of 30 patients with IC/BPS and 26 control subjects were enrolled in this study. The control subjects were younger (mean age, 32.4±1.56 years) than the IC/BPS patients (50.6±2.68 years). There were 26 women and 4 men in IC/BPS group, whereas 16 women and 10 men comprised the control group.

**Figure 1:**
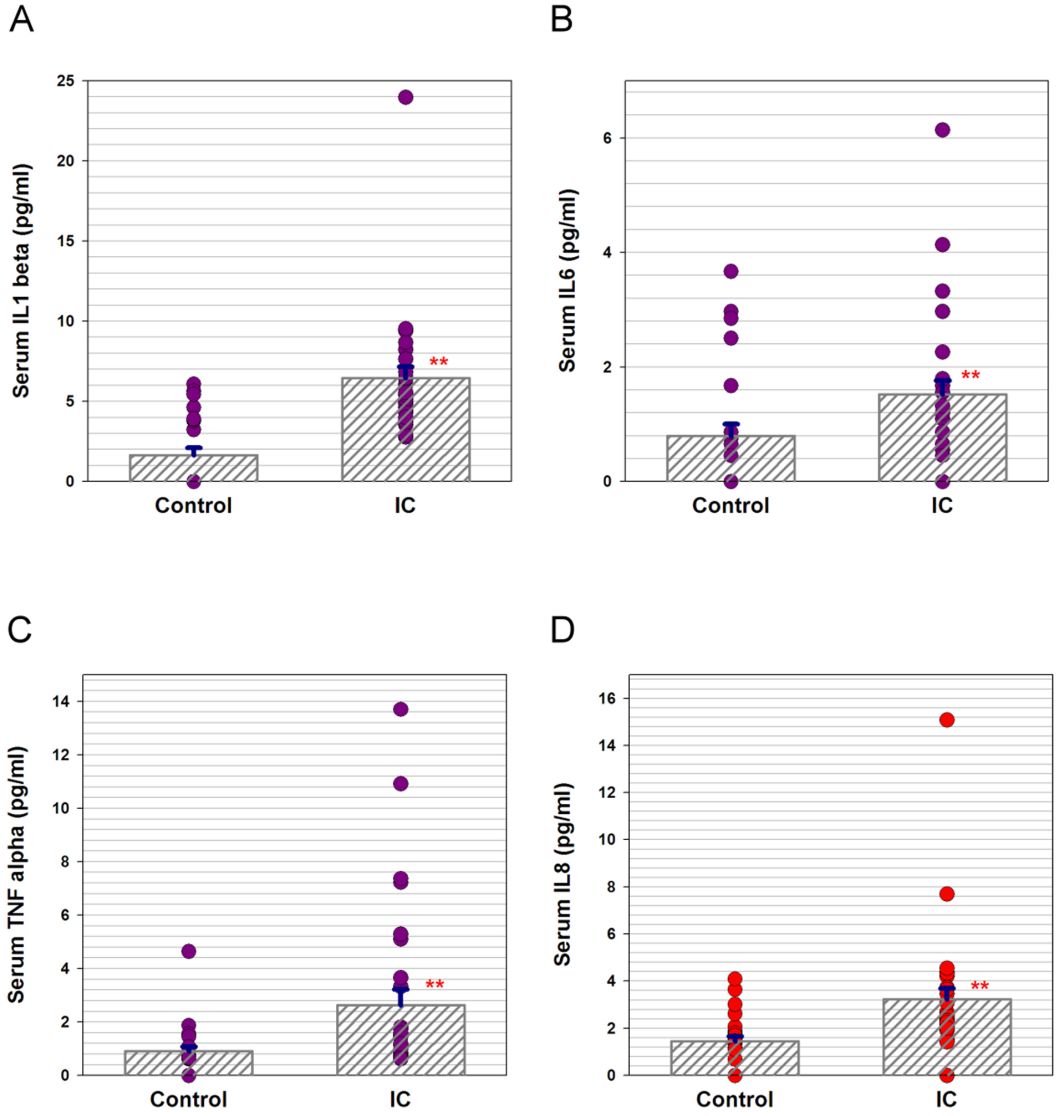
Scatter plots of serum (A) IL-1β, (B) IL-6, (C) TNF-α, and (D) IL-8 levels in IC/BPS patients and control subjects.

The mean serum CRP level was significantly greater in IC/BPS patients (*p* = 0.031), as was the mean serum NGF level (*p* = 0.015), compared to those of the control subjects. The pro-inflammatory cytokines/chemokine IL-1β, IL-6, TNF-α, and IL-8 levels were significantly higher in the patients with IC/BPS than in the control subjects (all *p*<0.001) (**Table 1** **,** **Fig. 1**). Because the age and gender distribution was not similar between two groups, we also analyzed the correlation of serum pro-inflammatory protein levels with age and gender. Table 2 shows the correlation coefficient data between serum pro-inflammatory protein levels and age or gender. The results revealed that only serum CRP and TNF-α were positively correlated with age, while the serum NGF, IL-1beta, IL-6 and IL8 all showed no significant difference with age in the control group. However, all serum pro-inflammatory protein levels were not associated with gender in the control group. All serum pro-inflammatory protein levels in IC/BPS group were not associated with age or gender.

**Table 1:** Expression of serum CRP, NGF, IL-1β, IL-6, TNF-α, and IL-8 in IC/BPS patients and controls.

	Controls (*n = *26)	IC/BPS (*n = *30)	*P value*	
CRP (mg/L)	0.61±0.07 (0.10–1.60)	1.72±0.49 (0.10–17.9)	0.031	
NGF (pg/mL)	1.90±0.38 (0.00–5.85)	3.48±0.55 (0.00–18.0)	0.015	
IL-1*β* (pg/mL)	1.64±0.47 (0.00–6.08)	6.45±0.71 (2.77–23.96)	<0.0001	
IL-6 (pg/mL)	0.79±0.21 (0.00–3.67)	1.52±0.24 (0.00–6.14)	<0.0001	
TNF-α(pg/mL)	0.91±0.17 (0.00–4.64)	2.63±0.60 (0.62–13.70)	<0.0001	
IL-8 (pg/mL)	1.45±0.21 (0.00–4.09)	3.23±0.48 (0.00–15.08)	<0.0001	

Mean ± standard error (min–max).

CRP: C-reactive protein, NGF: nerve growth factor, IL: interleukin, TNF-α: tumor necrosis factor-α, IC/BPS: interstitial cystitis/bladder pain syndrome.

**Table 2:** The correlation coefficient between serum CRP, NGF and cytokines/chemokines and age or gender in IC/BPS and control group.

Patients	Factor		CRP	NGF	IL-1*β*	IL-6	TNF-α	IL-8
Control	age	R	0.53	−0.06	0.22	0.06	0.50	0.12
		p value	0.01	0.76	0.28	0.76	0.01	0.56
	gender	R	0.22	0.17	0.22	0.08	0.07	0.35
		p value	0.27	0.38	0.24	0.68	0.70	0.06
IC/BPS	age	R	0.22	0.17	0.22	0.08	0.07	0.35
		p value	0.27	0.38	0.24	0.68	0.70	0.06
	gender	R	−0.11	0.05	−0.23	−0.10	−0.03	−0.09
		p value	0.5	0.78	0.23	0.59	0.89	0.62

CRP: C-reactive protein, NGF: nerve growth factor, IL: interleukin, TNF-α: tumor necrosis factor-α, IC/BPS: interstitial cystitis/bladder pain syndrome. R: correlation coefficient.

**Figure 2:**
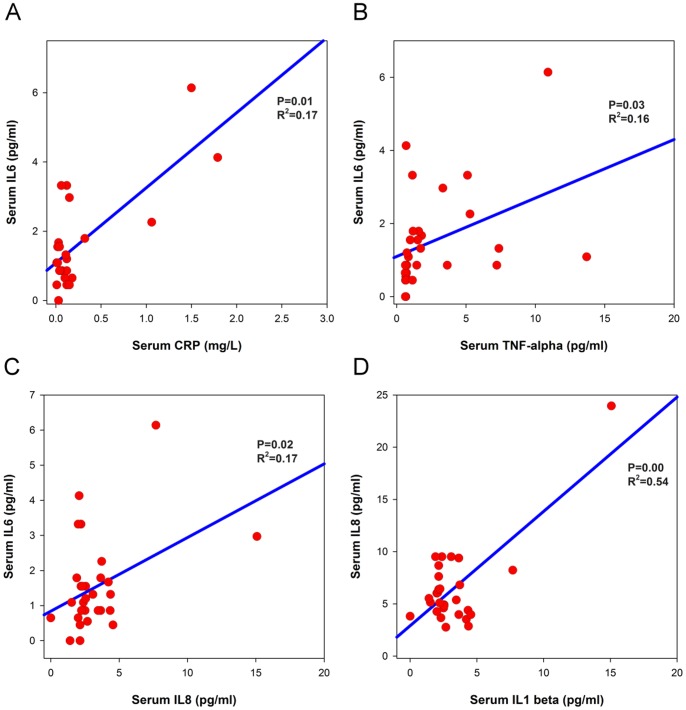
Significant correlations of (A) IL-1β and CRP, (B) IL-6 and TNF-α, (C) IL-6 and IL-8, and (D) IL-8 and IL-1β levels in sera of IC/BPS patients.

The analysis between pro-inflammatory cytokines, IL-8, and CRP in IC/BPS serum samples, showed significant association between IL-1β and IL-8 (**Fig. 2A**, r^2^ = 0.54, *p*<0.001), IL-6 and CRP (**Fig. 2B**, r^2^ = 0.17, *p = *0.01), IL-6 and IL-8 (r^2^ = 0.17, *p* = 0.02), and IL-6 and TNF-α (r^2^ = 0.16, *p = *0.03). The VAS score of the IC/BPS patients did not show any significant association with any of the measured protein.

## Discussion

This pilot study demonstrated increased pro-inflammatory cytokines and chemokine including IL-1β, IL-6, TNF-α, and IL-8, as well as serum CRP and NGF levels were observed in IC/BPS patients. Cytokines and chemokines play crucial roles in the pathogenesis of several chronic inflammatory diseases. Although increased urinary levels of these proteins have been reported, the up-regulated profiles of these cytokines/chemokine as well as CRP and NGF levels in serum suggest that IC/BPS is involved with systemic chronic inflammatory conditions.

Development of biomarkers for diagnosis of IC/BPS may be conducted through discovery based proteomics that identifies previously unrecognized proteins in tissues and biofluids of such patients, or to investigate the specific proteins linked to disease-associated processes. In addition to the urothelial defense molecules, inflammatory proteins in the urine have important roles in the pathogenesis of IC/BPS [10]. The general function of cytokines is inflammatory cell adhesion and infiltration of leukocytes into inflammatory foci. Cytokines measured in previous studies could originate in the urine from both resident and infiltrating cells, including leukocytes, epithelial cells, and detrusor smooth muscle cells [15]. Bladder biopsies of IC/BPS patients reported previously confirmed the involvement and presence of eosinophils and macrophages in the urothelium and mast cells in the detrusor muscle [16]. Involvement of eosinophils is also supported by urine cytology results showing increased urinary eosinophil cationic protein in the urine of IC/BPS patients [17]. Mast cells are considered might be the crucial effector cells for the immune response implicated in the pathogenesis of BPS/IC [8], [16].

Previous study of serum NGF levels in patients with IC/BPS revealed that serum NGF was elevated in IC/BPS patients compared with control subjects [18]. The clinical characteristics and medical co-morbidities did not show significant differences between IC/BPS patients with high and low serum NGF levels [18]. These results suggest that although chronic inflammation is involved in IC/BPS, the underlying pathophysiology for the phenotypical presentation might differ by IC/BPS subtype. Investigation of serum cytokines/chemokine levels could be helpful for differentiation among the phenotypes of IC/BPS.

Urine specimens from IC/BPS patients have significantly increased cytokine and chemokine levels, including IL-6 and IL-8 [12]. In the CYP-induced rat cystitis model, multiplex analysis of cytokine levels also revealed increased inflammation-associated cytokines in the urine and bladder tissue, including IL-6, IL-1β, monocyte chemotactic protein-1, and IL-10 [19]. Urine IL-6 and IL-8 levels are significantly elevated in IC/BPS patients. IC/BPS patients have a strong positive association between urinary IL-8 levels and bladder mast cell counts, however, IL-8 was not significantly different between patients with ulcerative and non-ulcerative IC/BPS [20]. The increased tissue cytokine and chemokine levels could induce afferent sensitization, which could result in bladder pain symptoms in IC/BPS patients [21]. A recent study investigating urinary chemokines between ulcerative and non-ulcerative IC/BPS revealed a significant fivefold to twentyfold increase in CXCL-10 and CXCL-1 and IL-6 and NGF in ulcerative type IC/BPS [22].

IL-1β is an important mediator of the inflammatory response and is involved in a variety of cellular activities, including cell proliferation, differentiation, and apoptosis [23]. Significant elevation of urinary interleukin-1β was found in patients with bacterial cystitis compared to patients with IC/BPS and control subjects [24]. Bladder infections are typically accompanied by an innate immune response involving vigorous IL-6 and IL-8 production [25]. Increased serum IL-6 and IL-8 in chronic cystitis might indicate an adaptive immune response after previous bladder infections. Elevated IL-1β, IL-6, and IL-8 could be used to differentiate patients with suspicious IC/BPS and inflammatory reactions after bacterial infection. The up-regulated profile of serum IL-1β, IL-6, TNF-α, and IL-8 levels in IC/BPS patients might potentially have a prognostic role and/or serve as a tool in choosing the proper therapeutic regimen for treatment. Increased expression of pro-inflammatory IL-1β, IL-6, TNF-α, and IL-8 in the sera of IC/BPS patients implies not only mast cell activation, but also some other inflammatory mediators play important roles in the pathogenesis of IC/BPS.

Increasing emphasis is being given to the role of urinary neurotrophins, namely NGF and brain derived neurotrophic factor, as key factors in urinary bladder dysfunctions such as OAB and IC/BPS. NGF acts as a local stress mediator in perceived stress and allergy and increased NGF messaging contributes to worsening of cutaneous inflammation [26]. In our study, serum NGF and CRP levels were significantly increased in IC/BPS patients, suggesting that chronic inflammation is involved in this bladder disorder. Irritable bowel syndrome, fibromyalgia, and chronic fatigue syndrome are more prevalent in patients with IC/BPS than in asymptomatic control subjects, and result in significant daily impact [27]. In conjunction with increased serum cytokines and chemokines in IC/BPS patients, we postulate that these circulating pro-inflammatory proteins could modulate sensory function in specific organs, including the urinary bladder, and cause characteristic IC symptoms. Treatment of IC/BPS based on this hypothesis might include anti-inflammatory agents in addition to local intravesical therapy.

IC/BPS is a heterogenous syndrome with different severity of inflammation and clinical presentations. The severe inflammation IC patients had trend to older at symptom onset, smaller bladder capacity under anesthesia, presence of mucosal cracks and increased bladder vascularity. Some urinary cytokine level, including IL-6 have been reported to increase in IC bladders with severe inflammation [11], [12]. In this study, although the serum cytokines/chemokine levels in IC/BPS group showed significantly increased compared with the controls, there were high variation of the inflammatory biomarkers, suggesting mixed groups with mild and severe inflammation or different pathophysiology might exist in our IC/BPS patients.

The main limitation of this study is the lack of age matched controls. Because most of the middle aged patients visiting the urology department had previous UTI, systemic diseases, or lower urinary tract symptoms, it was difficult for us to enroll the age match controls for comparison in this pilot study. A future extended study with large cohort of IC/BPS patients and age matched controls is mandatory.

## Conclusions

Increased serum pro-inflammatory cytokines and chemokines, including IL-1β, IL-6, TNF-α, and IL-8, as well as CRP and NGF levels were observed in IC/BPS patients. This implies not only mast cell activation, but also that some other inflammatory mediators could play important roles in the pathogenesis of IC/BPS, suggesting that IC/BPS has a chronic inflammatory component.
